# Attitudes toward and training in medications for opioid use disorders: a descriptive analysis among employees in the youth legal system and community mental health centers

**DOI:** 10.1186/s13011-024-00614-w

**Published:** 2024-06-21

**Authors:** Lauren M. O’Reilly, Katherine Schwartz, Steven A. Brown, Allyson Dir, Logan Gillenwater, Zachary Adams, Tamika Zapolski, Leslie A. Hulvershorn, Matthew Aalsma

**Affiliations:** 1 Department of Pediatrics, Indiana University School of Medicine, Indianapolis, IN USA; 2 Department of Biostatistics and Health Data Science, Indiana University School of Medicine, 401 W. 10th St, Indianapolis, IN 46202 USA; 3 Department of Psychiatry, Indiana University School of Medicine, Indianapolis, IN USA

**Keywords:** Medications for opioid use disorder, MOUD, Adolescent, Juvenile justice, Legal system, Community mental health, Acceptability, Effectiveness, Training

## Abstract

**Background:**

Research demonstrates gaps in medications for opioid use disorder uptake (MOUDs; methadone, buprenorphine, and naltrexone) especially among adolescents. These gaps may be partly attributable to attitudes about and training in MOUDs among youth-serving professionals. We extended prior research by conducting descriptive analyses of attitudes regarding effectiveness and acceptability of MOUDs, as well as training in MOUDs, among youth legal system (YLS) employees and community mental health center (CMHC) personnel who interface professionally with youth.

**Methods:**

Using survey data from participants (*n* = 181) recruited from eight Midwest counties, we examined: (1) differences in MOUD attitudes/training by MOUD type and (2) by respondent demographics, and (3) prediction of MOUD attitudes/training by participant-reported initiatives to implement evidence-based practices (EBPs), workplace culture around EBPs, and workplace stress. Attitudes and training were measured in reference to five MOUD types (methadone, oral buprenorphine, injectable buprenorphine, oral naltrexone, injectable naltrexone) on three subscales (effectiveness, acceptability, training).

**Results:**

Wilcoxon signed-rank tests demonstrated that most outcomes differed significantly by MOUD type (differences observed among 22 of 30 tests). Kruskal-Wallis tests suggested MOUD differences based on demographics. For methadone, CMHC providers endorsed greater perceived effectiveness than YLS providers and age explained significant differences in perceived effectiveness. For buprenorphine, CHMC providers viewed oral or injectable buprenorphine as more effective than YLS employees, respondents from more rural counties viewed oral buprenorphine as more effective than those from less rural counties, and age explained differences in perceived effectiveness. For naltrexone, perceived gender differed by gender. Hierarchical ordinal logistic regression analysis did not find an association between personal initiatives to implement EBPs, workplace culture supporting EBPs, or workplace stress and effectiveness or acceptability of MOUDs. However, personal initiatives to implement EBPs was associated with training in each MOUD.

**Conclusions:**

These results highlight a few key findings: effectiveness/acceptability of and training in MOUDs largely differ by MOUD type; setting, rurality, age, gender, and education explain group differences in perceived effectiveness of and training in MOUDs; and implementing EBPs is associated with training in MOUDs. Future research would benefit from examining what predicts change in MOUD attitudes longitudinally.

**Supplementary Information:**

The online version contains supplementary material available at 10.1186/s13011-024-00614-w.

## Introduction

Addressing opioid misuse and opioid use disorders among youth (i.e., 12–17 years old) is an urgent need. Rates of synthetic opioid overdose mortality increased nearly 3000% between 1999 and 2021 [1], and since 2020, opioid overdose mortality among adolescents increased at a higher rate than adults [2]. Research suggests that approximately 3.5% of youth (1.5 million youth) reported misusing prescription opioids (using one’s own medication in any way not directed by a doctor or using without a prescription) [3, 4]. Rates of opioid use disorder (OUD) diagnosis has increased 6-fold from 2001 to 2014 among 13- to 25-year-olds [5]. Medications for opioid use disorder (MOUDs)─methadone, buprenorphine, and naltrexone─are evidence-based treatments that have been shown to be safe and effective at reducing opioid-related and overall mortality, substance use, physical and psychological problems, and legal involvement in adults [6–8]. MOUDs are also associated with increased treatment retention [8]. Significantly less research on MOUDs has been conducted among youth compared to adults, yet buprenorphine, naltrexone, and methadone receipt have all been associated with improved treatment retention and reduced non-prescribed opioid use while on the medication [9, 10]. The American Academy of Pediatrics, American Academy of Child and Adolescent Psychiatry, and Society for Adolescent Health and Medicine have endorsed MOUDs for adolescents.

Despite these recommendations, youth access is low [11, 12]. A study of adolescents with a documented OUD diagnosis and connected with primary care found that 86% of 16- to 17-year-olds did not receive an MOUD [13]. Youth admitted to substance use treatment services are less likely to have MOUDs included as part of their treatment plan compared to adults (2% vs. 93%) [14]. Further, a review of 160 residential substance use treatment centers that provide youth services found that only a quarter offered MOUDs [15]. While not specific to youth, research has also found that those who interact with the legal system are unlikely to receive MOUDs when diagnosed with OUD [12].

Previous research has examined attitudes toward MOUDs among various groups (e.g., physicians, people who have an OUD history). Across studies, positive attitudes toward the acceptability and effectiveness of MOUDs were associated with more knowledge about MOUDs, more frequent interaction with patients receiving MOUDs, and less stigmatizing beliefs about substance use disorders [16]. Among both prescribing and non-prescribing healthcare providers, those with more exposure to pharmacological interventions had more positive MOUD attitudes [16]. Exposure to MOUDs may be related to more specialized training and increased knowledge about their effectiveness, thereby increasing their acceptability. Among providers and the general public, greater endorsement of abstinence-only treatment philosophies (which extends to MOUDs) is negatively associated with MOUD acceptability [16, 17], and greater endorsement of viewing OUD as an illness was positively associated with beliefs about MOUD effectiveness [18]. Importantly, attitudes appear to differ by MOUD and profession. For example, physicians rated buprenorphine as more effective than methadone (contrary to the robust literature supporting methadone) [19], which was rated as more effective than naltrexone in treatment for OUD [20]. Medical professionals also held more positive views toward MOUDs compared to health care support staff [21]. Outside the health profession, US legal system personnel who endorsed stigmatizing beliefs toward legal-involved individuals endorsed more negative MOUD attitudes [22].

Closely tied to attitudes and knowledge about MOUDs is training in MOUDs. Physicians who have received training in addiction treatment or work closely with those with specialized training endorse more positive attitudes toward MOUDs [16]. Additionally, physicians rated both their knowledge about MOUDs and likelihood to prescribe MOUDs as higher following MOUD waiver training in medical school [23]. Substance use treatment counselors were also more likely to rate buprenorphine as effective if they received buprenorphine-specific training [24].

MOUD attitudes and training at the individual level need to be contextualized within workplace-related factors, which can facilitate or impede MOUD uptake. For example, physicians’ perception of their emergency department as having an innovative climate was associated with MOUD support [25]. Less emphasis on 12-step models in substance use treatment centers was associated with greater counselor-rated MOUD acceptability and effectiveness [26]. Within the adult legal system, rehabilitation-oriented agencies were more supportive of MOUDs [27]. Organizational culture surrounding attitudes toward evidence-based practice (EBP) likely extend to MOUDs, as negative attitudes toward EBPs could correspond to lower support or administration of MOUDs, consistent with an extensive literature linking such attitudes with minimal EBP uptake [28]. Organizational culture may also be captured through occupational stress, and prior research has demonstrated that workplace-related stress was associated with less interest in EBP adoption [29]. Considering associations between demographic and workplace-related factors and MOUD attitudes and training may help identify intervention targets to ultimately increase use of evidence-based substance use treatment for youth.

Prior research has guided our understanding of how various groups view MOUDs; however, three significant gaps remain. First, research has largely been conducted among medical health providers (i.e., physicians, nurses, pharmacists), with less research attention paid to other professionals from other systems. Among mental health providers broadly, research has focused on those working in substance use treatment centers (e.g., social workers, counselors, psychologists, peer recovery coaches) [30]. However, there is a significant gap in knowledge about MOUD attitudes and training among those who work in community mental health centers (CMHC) [31]. In addition to the lack of research among CMHC providers, there is limited research among professionals within the youth legal system (YLS). One review found support for the pervasiveness of negative attitudes toward MOUDs in the criminal justice system broadly [32] but this has not been extended to the YLS. Second, research has been predominantly carried out among people who serve adults, and attitudes and experiences of youth-serving professionals have been neglected. Welsh and colleagues (2022) found that substance use treatment staff were less likely to support MOUD use in adolescents compared to adults; research specifically focused on employees who interface professionally with youth is needed. Third, most research has focused on buprenorphine and methadone, with less research available on naltrexone. Despite higher youth prescription rates of buprenorphine compared to naltrexone [5], naltrexone continues to be prescribed to youth. A scoping review of 152 studies examining MOUD perceptions among patients and providers included 63 about buprenorphine, 115 about methadone, and 16 about naltrexone, demonstrating the considerable gap in attitudinal research about naltrexone [33]. This review included research from outpatient and inpatient substance use treatment programs, prisons, and primary care offices, emphasizing that naltrexone attitudes and training are not well understood among CMHC and YLS employees.

Taken together, focusing on CMHC and YLS professionals is crucial in understanding potential upstream effects on youth substance use treatment. CMHC and YLS professionals may affect whether youth are referred for treatment [34], as well as youth attitudes toward MOUDs, willingness to engage in treatment, and adherence to medication once prescribed [26, 31, 35].

The current paper aims to address gaps in the literature by conducting a descriptive analysis of MOUD attitudes and training among youth-interfacing CMHC and YLS professionals. First, while research has traditionally examined MOUDs separately, it remains an empirical question whether MOUD attitudes or training differ by MOUD (Aim 1). Second, given the need to further explore group differences in MOUD attitudes and training, we examined whether demographic information explained group differences in MOUD attitudes and training (Aim 2). Third, we examined the association between MOUD attitudes and training with demographic variables and workplace-related variables in hierarchical regression models. While the aims were exploratory, we hypothesized that methadone would be viewed less favorably compared to buprenorphine and naltrexone, CMHC employees would have more positive attitudes toward and more training in MOUDs compared to YLS employees, and endorsing a workplace culture supportive of EBPs would be associated with favorable MOUD attitudes and more training.

## Materials and methods

### Sample

The current sample was derived from the Alliances to Disseminate Addiction Prevention and Treatment (ADAPT) project in eight counties in a Midwest state [36]. The primary aim was to study the implementation of a learning health system between the YLS and CMHCs to increase legal-involved-youth connection to evidence-based substance use treatment. ADAPT was preregistered and approved by the author’s Institutional Review Board (Protocol #1910282231). Ethical standards were in accordance with the Declaration of Helsinki.

As part of this study, surveys of YLS and CMHC personnel were collected at five waves from 2020 to 2023 administered approximately every seven months. Subjects were identified through publicly available staff rosters, organization charts, or agency lists of contact information. Those recruited for study participation included frontline staff (i.e., probation officers, therapists, skills trainers), staff supervisors, and agency decisionmakers. Potential subjects were contacted to participate via email. We utilized data from the first wave of data collection to avoid potential changes in attitudes due to project involvement. There were 227 individuals (71 [31.3%] YLS and 156 [68.7%] CMHC) who were considered eligible and sent an invitation to participate in the first wave survey. Of those eligible, 37 (16.3%) were excluded due to no response (*n* = 36 [5 YLS and 31 CMHC]) or declined to participate (*n* = 1 [YLS]); 190 individuals (83.7%) were enrolled at baseline. Individuals provided informed consent to voluntarily participate in data collection; nine individuals did not provide affirmative consent resulting in a final analytical sample of 181 individuals. Sample characteristics are found in Appendix 1. Data are not made publicly available to protect study participant privacy. Data analytic code will be made available upon request.

## Measures

### Demographic predictors

The following respondent demographic information was collected: age (18–25, 26–35, 36–45, 46–55, 56–65 years old, 66 or older), race (White, Black/African American, American Indiana or Alaska Native, Asian, Native Hawaiian or Pacific Islander, Other), ethnicity (Hispanic/Latinx, Non-Hispanic/Non-Latinx, do not know), gender (female, male, transgender, nonbinary), time in position (less than one year, 1–4 years, 5–9 years, 10–14 years, 15–19 years, 20 or more years), highest education (high school, some college, Associate’s, Bachelor’s, Master’s, doctorate), job satisfaction (very dissatisfied, dissatisfied, not satisfied or dissatisfied, satisfied, very satisfied), rurality of the county in which they work (more versus less rural), and county in which they work. For rurality, we utilized the Purdue University Index of Relative Rurality from 2010 [37] with a cutoff of 0.42; less rural counties’ IRR scores ranged from 0.40 to 0.42 and more rural counties ranged from 0.45 to 0.53.

### Workplace-related predictors: endorsement of evidence-based practices

#### Endorsement of evidence-based practices: Implementation Citizenship Behavior Scale (ICBS) and Implementation Climate Scale (ICS)

Developed by Ehrhart and colleagues (2015), the ICBS is a six-item questionnaire that measures employees’ support and pursuit of EBP implementation within two domains: helping others (e.g., “Assist others to make sure they implement evidence-based practices properly”) and remaining informed on EBPs (e.g., “Keeping informed of changes in evidence-based practice policies and procedures”). Response options are measured on a 5-point Likert from 0 (not at all) to 4 (frequently, if not always), and items were averaged. In the current sample, Cronbach’s alpha was 0.86.

Developed by Ehrhart and colleagues (2014), the ICS is an 18-item measure of organizational climate of EBP implementation along six subscales: (1) focus on EBP (e.g., “One of this team’s main goals is to use evidence-based practice effectively”), (2) educational support for EBP (e.g., “This team provides conferences, workshops, or seminars focusing on evidence-based practices”), (3) recognition for EBP (e.g., “Staff on this team who use evidence-based practice are seen as experts”), (4) rewards for EBP (e.g., “This team provides financial incentives for the use of evidence-based practices”), (5) selection for EBP (e.g., “This team selects staff who have had formal education supporting evidence-based practice”), and (6) selection for openness (e.g., “This team selects staff open to new types of interventions”). Response options were scored on a 5-point Likert from 0 (not at all) to 4 (very great extent), and all items were averaged. In the current sample, Cronbach’s alpha was 0.93.

### Endorsement of workplace stress: Texas Christian University Systems of Organizational Functioning Survey

The Texas Christian University Institute of Behavioral Research developed the Systems of Organizational Functioning survey [38, 39], which is a 162-item measure comprised of 10 scales that index a wide range of organizational factors including readiness for change, job attitudes, workplace factors, and organizational climate. The Texas Christian University Institute of Behavioral Research developed the Systems of Organizational Functioning survey [38, 39], which is a 162-item measure comprised of 10 scales that index organizational factors including readiness for change, job attitudes, workplace factors, and organizational climate. The stress subscale was utilized, which is a four-item measure capturing workplace stress and pressures (e.g., “Staff members are under too many pressures to do their jobs effectively”). Respondents were asked rate their agreement with the statements on a 5-point Likert scale from 1 (strongly disagree) to 5 (strongly agree). The items were averaged for analysis. In the current sample, Cronbach’s alpha was 0.92.

### Primary outcome

#### Attitudes toward MOUD treatment

Survey respondents were asked to rate the perceived effectiveness and acceptability of MOUDs, as well as their training in MOUDs. For effectiveness, respondents were prompted by the following question, “Based on your knowledge and personal experience, to what extent do you consider each of the following medications for opioid use disorder to be effective with justice involved populations?” Response options ranged from 1 (not at all effective) to 7 (very effective). For acceptability, respondents were prompted by the following question, “In your opinion, how acceptable is each of the following medications for the treatment of opioid use disorder with justice involved populations?” Response options ranged from 1 (completely unacceptable) to 7 (very acceptable). For training, respondents were prompted by the following question, “To what extent have you received specific training about the following?” Response options ranged from 0 (no training) to 7 (extensive training). Refer to Appendix 2 for univariate statistics of each item. Therefore, all questions were not specifically referring to MOUDs for adolescents. For each scale (i.e., effectiveness, acceptability, and training), respondents answered questions corresponding to five MOUDs: methadone, oral buprenorphine (Suboxone®), monthly (extended-release) injection buprenorphine (Sublocade®), oral naltrexone, and monthly injection naltrexone (Vivitrol®).

### Analysis

To examine differences by MOUD type (Aim 1), we performed Wilcoxon signed-rank tests, which is a nonparametric test to compare median differences of ordinal variable distributions derived from the same sample. To determine differences in MOUD outcomes by demographic variables (Aim 2), we conducted Kruskal-Wallis tests. For each analysis, a complete case sample was used.

For Aim 3, we pursued multiple imputation to increase sample size given the numerous variables included in the multivariable models. First, we removed individuals who were missing demographic information (i.e., age, race, ethnicity, gender, time at position, job satisfaction, highest education level, and county; *n* = 5). Second, we required at least 20% data availability by each MOUD subscale (i.e., at least one MOUD item response) and, therefore, removed those who were missing all MOUD items when rating effectiveness, acceptability, and training (*n* = 69) to avoid imputing an entire subscale. The final sample size was 105 respondents (sample characteristics found in Appendix 1). Third, we conducted multiple imputation with 10 imputations (for a total of 1,050 analyzable observations) using the fully conditional specification to impute categorical response options for all 15 MOUD item-level outcomes [40]. Fourth, we predicted MOUD outcomes from the ICBS, ICS, and stress scale (Aim 3). We conducted hierarchical ordinal logistic regression with a random effect for system (YLS vs. CMHC) nested within county. Multinomial distribution and cumulative logit options were specified. Demographic variables (i.e., age, race, ethnicity, education level, time at position, job satisfaction, and rurality) were included as covariates, as well as fixed effects of system. All analyses were conducted in SAS 9.4 [41].

## Results

For Aim 1, we compared MOUDs by each subscale using Wilcoxon signed-rank tests (Table 1). Results suggested that perceived effectiveness of MOUDs differed significantly by type of MOUD except for the following comparisons: oral buprenorphine versus injectable buprenorphine, and oral buprenorphine versus oral naltrexone. For acceptability, the following comparisons were not statistically significantly different: oral buprenorphine versus injectable buprenorphine, oral buprenorphine versus oral naltrexone, and injectable buprenorphine versus oral naltrexone. Finally, for training, the following comparisons were not statistically significantly different: methadone versus oral buprenorphine, methadone versus injectable naltrexone, and oral naltrexone versus injectable naltrexone.

**Table 1:** Wilcoxon signed-rank test comparing MOUD attitudes by MOUD type

	Effectiveness			
	*N*	M (SD)	S-value	*p*-value
Methadone vs. Buprenorphine (oral)	114	-0.68 (1.20)	-555	< 0.001
Methadone vs. Buprenorphine (inject)	96	-0.77 (1.27)	-523	< 0.001
Methadone vs. Naltrexone (oral)	97	-0.42 (1.61)	-242	0.006
Methadone vs. Naltrexone (inject)	100	-0.99 (1.90)	-657.5	< 0.001
Buprenorphine (oral) vs. Buprenorphine (inject)	96	-0.08 (1.04)	-39.5	0.532
Buprenorphine (oral) vs. Naltrexone (oral)	98	-0.17 (1.37)	101.5	0.213
Buprenorphine (oral) vs. Naltrexone (inject)	101	-0.35 (1.65)	-271	0.027
Buprenorphine (inject) vs. Naltrexone (oral)	91	0.27 (1.35)	168	0.040
Buprenorphine (inject) vs. Naltrexone (inject)	93	-0.28 (1.31)	-114.5	0.014
Naltrexone (oral) vs. Naltrexone (inject)	96	-0.56 (1.04)	-318.5	< 0.001
	**Acceptability**			
Methadone vs. Buprenorphine (oral)	121	-0.50 (1.22)	-334	< 0.001
Methadone vs. Buprenorphine (inject)	112	-0.63 (1.37)	-359.5	< 0.001
Methadone vs. Naltrexone (oral)	109	-0.62 (1.59)	-324	< 0.001
Methadone vs. Naltrexone (inject)	114	-0.92 (1.77)	-509.5	< 0.001
Buprenorphine (oral) vs. Buprenorphine (inject)	111	-0.09 (0.94)	-53.5	0.329
Buprenorphine (oral) vs. Naltrexone (oral)	109	-0.11 (1.23)	-35.5	0.468
Buprenorphine (oral) vs. Naltrexone (inject)	113	-0.40 (1.46)	-240.5	0.003
Buprenorphine (inject) vs. Naltrexone (oral)	105	-0.02 (1.22)	6	0.928
Buprenorphine (inject) vs. Naltrexone (inject)	108	-0.28 (1.14)	-90.5	0.010
Naltrexone (oral) vs. Naltrexone (inject)	109	-0.28 (0.87)	-78	0.001
	**Training**			
Methadone vs. Buprenorphine (oral)	144	-0.11 (0.95)	-96	0.092
Methadone vs. Buprenorphine (inject)	144	0.40 (1.28)	290	< 0.001
Methadone vs. Naltrexone (oral)	144	0.26 (1.36)	169	0.031
Methadone vs. Naltrexone (inject)	143	0.15 (1.33)	103	0.258
Buprenorphine (oral) vs. Buprenorphine (inject)	143	0.52 (1.17)	290.5	< 0.001
Buprenorphine (oral) vs. Naltrexone (oral)	142	0.37 (1.25)	219.5	< 0.001
Buprenorphine (oral) vs. Naltrexone (inject)	142	0.26 (1.23)	155	0.016
Buprenorphine (inject) vs. Naltrexone (oral)	143	-0.15 (1.25)	-102.5	0.049
Buprenorphine (inject) vs. Naltrexone (inject)	142	-0.25 (1.26)	-162.5	0.008
Naltrexone (oral) vs. Naltrexone (inject)	142	-0.11 (0.77)	-30.5	0.086

*p < 0.05 *p < 0.01

In addition to examining whether the comparison tests were significant, the direction of the Wilcoxon signed-rank test also demonstrated important findings. Among the comparison tests that were statistically significant, methadone was viewed as less effective and acceptable compared to every other MOUD. When comparing buprenorphine and naltrexone, oral route of administration was viewed as less effective and acceptable compared to injectable route of administration. Specifically, oral buprenorphine was rated as less effective and acceptable than injectable naltrexone, and injectable buprenorphine was viewed as more effective than oral naltrexone. However, when comparing injectable buprenorphine and injectable naltrexone, buprenorphine was viewed as less effective and acceptable (Table 1).

For training in MOUDs, respondents reported more training in oral buprenorphine compared to all other MOUDs (Table 1), followed by methadone which had greater training than injectable buprenorphine and naltrexone. Respondents rated less training in injectable buprenorphine than naltrexone and less training in oral naltrexone than injectable naltrexone.

For Aim 2, we conducted a series of Kruskal-Wallis tests to examine whether demographic variables differed by MOUD item. When examining effectiveness of MOUDs (Table 2), we found group differences in perceived MOUD effectiveness based on system (i.e., YLS vs. CMHC), rurality, age, gender, and education level. There was a significant difference between YLS and CMHC respondents for methadone (χ^2^ = 4.13 [1, *p* = 0.04]), oral buprenorphine (χ^2^ = 10.77 [1, *p* < 0.01]), and injectable buprenorphine (χ^2^ = 5.48 [1, *p* = 0.02]); CMHC respondents rated all as more effective than YLS respondents. Respondents from more rural counties rated oral buprenorphine as more effective than those from less rural counties (χ^2^ = 3.92 [1, *p* = 0.05]). Significant differences by age were observed for methadone (χ^2^ = 12.28 [5, *p* = 0.03]), oral buprenorphine (χ^2^ = 13.85 [5, *p* = 0.02]), and injectable buprenorphine (χ^2^ = 17.29 [5, *p* < 0.01]). Post hoc pairwise two-sided comparisons demonstrated that 46- to55-year-olds rated methadone as less effective than 36- to 45-year-olds (Wilcoxon Z = -2.96, *p* = 0.04), and 36- to 45-year-olds rated injectable buprenorphine as more effective than 56- to 65-year-olds (Wilcoxon Z = 2.96, *p* = 0.04). Significant differences in injectable naltrexone were based on gender (χ^2^ = 6.91 [2, *p* = 0.03]), and finally, differences in oral buprenorphine were observed by education level (χ^2^ = 15.9 [4, *p* < 0.01])

**Table 2:** Results from Kruskal-Wallis Tests of Group Differences in Effectiveness of MOUDs by Demographic Variables

Variable	Methadone^a^		Bup (oral)^b^		Bup (inject)^c^		Nal (oral)^d^		Nal (inject)^e^	
	Mean of ranks sums (*N*)	(χ^2^, df, *p*-value)	Mean of ranks sums (*N*)	(χ^2^, df, *p*-value)	Mean of ranks sums (*N*)	(χ^2^, df, *p*-value)	Mean of ranks sums (*N*)	(χ^2^, df, *p*-value)	Mean of ranks sums (*N*)	(χ^2^, df, *p*-value)
**System**										
YLS	52.3 (36)	**4.13 (1), ** ***p*** ** = 0.042**	46 (36)	**10.77 (1), ** ***p*** ** = 0.001**	40 (29)	**5.48 (1), ***p* = 0.019	42.1 (28)	3.58 (1), *p* = 0.058	48.4 (32)	0.79 (1), *p* = 0.373
CMHC	65.9 (82)		67.8 (80)		54.1 (66)		53.6 (68)		53.8 (68)	
**Rurality**										
Less rural	54.2 (27)	1.35 (1), *p* = 0.245	48.5 (25)	**3.92 (1), ** ***p*** ** = 0.048**	41.0 (21)	2.42 (1), *p* = 0.120	50.9 (21)	0.16 (1), *p* = 0.692	44.0 (23)	2.10 (1), *p* = 0.147
More rural	62.6 (90)		62.9 (90)		51.0 (72)		48.4 (73)		53.4 (75)	
**Age**										
18–25	56.9 (7)	**12.28 (5), ** ***p*** ** = 0.031**	41.8 (7)	**13.85 (5), ** ***p*** ** = 0.017**	12.5 (3)	**17.29 (5), ** ***p*** ** = 0.004**	17.2 (3)	9.89 (5), *p* = 0.079	10.8 (3)	8.48 (5), *p* = 0.132
26–35	63.1 (32)		65.2 (32)		51.6 (25)		48.7 (24)		48.3 (26)	
36–45	73.3 (37)		72.2 (35)		59.3 (42)		55.3 (30)		58.6 (31)	
46–55	44.5 (22)		48.2 (22)		41.0 (17)		56.7 (20)		52.2 (21)	
56–65	49.6 (10)		41.0 (10)		25.6 (7)		31.6 (9)		48.8 (9)	
66+	70.4 (4)		63.0 (4)		59.5 (4)		53.9 (4)		59.3 (4)	
**Gender**										
Male	60.9 (96)	3.58 (2), *p* = 0.167	60.0 (92)	0.35 (2), *p* = 0.838	49.9 (73)	5.07 (2), *p* = 0.079	51.0 (78)	4.95 (2), *p* = 0.084	54.8 (80)	**6.91 (2), ** ***p*** ** = 0.032**
Female	60.1 (21)		61.2 (23)		43.8 (19)		42.0 (16)		38.7 (18)	
Other	107.8 (2)		74.3 (2)		82.3 (3)		86.3 (2)		80.5 (2)	
**Race**										
White	61.9 (112)	0.21 (1), *p* = 0.650	60.3 (110)	0.07 (1), *p* = 0.794	50.4 (89)	0.95 (1), *p* = 0.329	50.2 (90)	0.06 (1), *p* = 0.804	53.3 (94)	2.06 (1), *p* = 0.151
Racial minority	56.7 (10)		63.2 (10)		40.8 (9)		47.8 (9)		38.6 (9)	
**Ethnicity**										
Non-Hispanic	60.3 (114)	0.15 (1), *p* = 0.702	59.0 (110)	0.49 (1), *p* = 0.486	48.4 (91)	0.01 (1), *p* = 0.933	50.1 (93)	0.73 (1), *p* = 0.392	51.3 (97)	0.08 (1), *p* = 0.782
Hispanic	54.3 (5)		49.3 (6)		49.5 (5)		39.1 (5)		55.0 (5)	
**Time at Position**										
<1 year	66.2 (11)	1.52 (3), *p* = 0.678	70.4 (14)	3.48 (3), *p* = 0.323	60.5 (10)	1.77 (3), *p* = 0.621	56.2 (9)	1.59 (3), *p* = 0.663	57.7 (43)	1.74 (3), *p* = 0.628
1–4 years	63.3 (67)		60.5 (63)		48.6 (44)		47.1 (45)		48.5 (44)	
5–9 years	60.3 (17)		63.2 (16)		48.5 (13)		55.0 (14)		55.6 (13)	
10 + years	53.7 (18)		50.1 (18)		46.9 (14)		50.9 (16)		55.5 (18)	
**Education**										
Some college	50.3 (3)	8.98 (4), *p* = 0.062	86.7 (3)	**15.9 (4), ** ***p*** ** = 0.003**	40.5 (2)	7.85 (4), *p* = 0.097	58.0 (2)	7.39 (4), *p* = 0.117	69.0 (2)	5.67 (4), *p* = 0.225
Bachelor’s	54.2 (45)		48.0 (46)		43.1 (47)		44.3 (48)		47.5 (39)	
Associate’s	115.5 (2)		110.0 (2)		88.5 (2)		93.0 (2)		92.0 (2)	
Master’s	65.7 (49)		67.8 (49)		54.5 (40)		53.6 (39)		54.0 (40)	
Doctorate	71.3 (10)		68.9 (10)		45.8 (6)		47.6 (7)		51.6 (8)	
**Job Satisfaction**										
Dissatisfied	65.5 (6)	0.09 (2), *p* = 0.955	67.4 (6)	2.54 (2), *p* = 0.281	62.8 (5)	1.36 (2), *p* = 0.506	64.5 (5)	1.57 (2), *p* = 0.457	67.2 (5)	2.78 (2), *p* = 0.249
Neutral	60.4 (50)		75.0 (43)		45.1 (9)		45.8 (10)		41.0 (10)	
Satisfied	103 (61.4)		58.6 (103)		49.2 (84)		49.6 (84)		52.4 (88)	

*Note p* < 0.05 is bolded. Mean scores are derived from sum of rank sums/N. ^a^Based on 122 individuals. ^b^Based on 120 individuals. ^c^Based on 98 individuals. ^d^Based on 99 individuals. ^e^Based on 103 individuals

When examining acceptability, there were no statistically significant group differences based on demographic groups (Appendix 3).

When examining training in MOUDs, age and education level were associated with group differences (Table 3). We found that endorsement of training for methadone (χ^2^ = 20.16 [5, *p* < 0.01]), oral buprenorphine (χ^2^ = 11.90 [5, *p* = 0.04]), and injectable buprenorphine (χ^2^ = 13.65 [5, *p* = 0.02]) differed by age. Post hoc pairwise two-sided comparisons demonstrated that for methadone, 46-55-year-olds endorsed less training than 36-45-year-olds (Wilcoxon Z = -2.96, *p* = 0.04) and 36-45-year-olds endorsed more training than 18-25-year-olds (Wilcoxon Z = 3.18, *p* = 0.02). Additionally, 18-25-year-olds endorsed less injectable buprenorphine training than those older than 65 (Wilcoxon Z = -2.94, *p* = 0.04). When examining by education level, we observed a significant difference in training for oral buprenorphine (χ^2^ = 15.71 [4, *p* < 0.01]). Post hoc pairwise two-sided comparisons demonstrated those with a Master’s degree had more training than those with a Bachelor’s (Wilcoxon Z = 3.00, *p* = 0.02).

**Table 3:** Results from Kruskal-Wallis Tests of Group Differences in Training in MOUDs by Demographic Variables

Variable	Methadone^a^		Bup (oral)^b^		Bup (inject)^c^		Nal (oral)^d^		Nal (inject)^e^	
	Mean of ranks sums (*N*)	(χ^2^, df, *p*-value)	Mean of ranks sums (*N*)	(χ^2^, df, *p*-value)	Mean of ranks sums (*N*)	(χ^2^, df, *p*-value)	Mean of ranks sums (*N*)	(χ^2^, df, *p*-value)	Mean of ranks sums (*N*)	(χ^2^, df, *p*-value)
**System**										
YLS	62.9 (37)	0.02 (1), *p* = 0.895	59.7 (35)	0.36 (1), *p* = 0.550	51.9 (42)	1.32 (1), *p* = 0.251	51.5 (32)	1.05 (1), *p* = 0.305	56.3 (33)	0.36 (1), *p* = 0.546
CMHC	63.8 (85)		63.8 (85)		59.4 (77)		58.1 (76)		60.3 (80)	
**Rurality**										
Less rural	73.8 (30)	0.04 (1), *p* = 0.838	68.6 (30)	0.22 (1), *p* = 0.637	75.4 (30)	0.44 (1), *p* = 0.507	71.4 (30)	0 (1), *p* = 0.984	70.1 (30)	0.02 (1), *p* = 0.881
More rural	72.1 (111)		72.4 (109)		70.3 (109)		71.5 (109)		71.3 (108)	
**Age**										
18–25	46.8 (50)	**20.16 (5), ** ***p*** ** = 0.001**	51.2 (50)	**11.90 (5), ** ***p*** ** = 0.036**	49.0 (50)	**13.65 (5), ** *p* ** = 0.018**	52.2 (50)	9.87 (5), *p* = 0.079	49.9 (50)	8.45 (5), *p* = 0.133
26–35	71.5 (41)		69.9 (41)		71.6 (41)		65.3 (41)		67.7 (41)	
36–45	90.2 (37)		83.1 (35)		80.5 (35)		84.8 (36)		80.2 (35)	
46–55	59.9 (26)		65.5 (26)		65.9 (26)		72.4 (26)		70.2 (25)	
56–65	73.7 (50)		75.7 (50)		77.0 (50)		77.2 (51)		84.8 (50)	
66+	123.7 (3)		121.7 (3)		128.8 (3)		91.5 (3)		89.5 (3)	
**Gender**										
Male	72.0 (115)	1.82 (2), *p* = 0.403	69.4 (113)	4.16 (2), *p* = 0.125	71.5 (114)	4.84 (2), *p* = 0.089	70.9 (115)	4.07 (2), *p* = 0.131	69.5 (114)	4.50 (2), *p* = 0.106
Female	76.4 (25)		81.4 (25)		71.5 (24)		74.4 (23)		78.0 (23)	
Other	102.7 (3)		106.7 (3)		121.5 (3)		116.7 (3)		114.0 (3)	
**Race**										
White	74.0 (136)	0.27 (1), *p* = 0.605	72.4 (134)	0.02 (1), *p* = 0.884	72.6 (134)	0.02 (1), *p* = 0.886	72.4 (134)	0.03 (1), *p* = 0.870	72.2 (133)	0.05 (1), *p* = 0.825
Racial minority	67.1 (10)		74.3 (10)		70.8 (10)		74.5 (10)		69.4 (10)	
**Ethnicity**										
Non-Hispanic	72.7 (135)	0.76 (1), *p* = 0.385	71.6 (133)	0.63 (1), *p* = 0.429	71.3 (133)	0.17 (1), *p* = 0.682	71.3 (133)	0.13 (1), *p* = 0.722	71.1 (132)	0.53 (1), *p* = 0.465
Hispanic	60.1 (8)		60.3 (8)		65.6 (8)		66.3 (8)		60.9 (8)	
**Time at Position**										
<1 year	79.3 (18)	1.33 (3), *p* = 0.721	82.2 (17)	2.42 (3), *p* = 0.490	69.4 (17)	0.27 (3), *p* = 0.965	68.5 (18)	2.27 (3), *p* = 0.518	74.2 (18)	2.42 (3), *p* = 0.490
1–4 years	71.2 (77)		68.2 (77)		72.1 (77)		69.6 (77)		67.4 (76)	
5–9 years	79.8 (19)		77.9 (18)		75.0 (18)		82.5 (18)		79.6 (17)	
10 + years	70.5 (23)		73.4 (23)		74.2 (23)		76.4 (22)		77.8 (23)	
**Education**										
Some college	90.3 (3)	6.96 (4), *p* = 0.138	131.0 (3)	**15.71 (4), ** ***p*** ** = 0.003**	98.2 (3)	8.69 (4), *p* = 0.069	87.8 (2)	2.63 (4), *p* = 0.621	103.5 (3)	6.37 (4), *p* = 0.173
Bachelor’s	64.7 (73)		61.0 (71)		63.6 (72)		67.6 (73)		64.6 (71)	
Associate’s	86.0 (2)		83.0 (2)		88.3 (2)		87.8 (2)		86.0 (2)	
Master’s	81.8 (59)		82.0 (59)		81.8 (58)		77.1 (58)		78.5 (58)	
Doctorate	82.1 (9)		79.2 (9)		71.2 (9)		75.9 (9)		75.1 (9)	
**Job Satisfaction**										
Dissatisfied	68.9 (7)	1.23 (2), *p* = 0.542	69.3 (7)	0.56 (2), *p* = 0.755	79.2 (7)	0.76 (2), *p* = 0.683	79.3 (7)	0.96 (2), *p* = 0.620	74.9 (7)	1.09 (2), *p* = 0.579
Neutral	84.3 (12)		79.7 (12)		79.2 (12)		80.3 (12)		81.7 (12)	
Satisfied	72.4 (124)		71.8 (122)		71.3 (122)		71.1 (122)		70.6 (121)	

*Note p* < 0.05 is bolded. Mean scores are derived from sum of rank sums/N. ^a^Based on 146 individuals. ^b^Based on 144 individuals. ^c^Based on 143 individuals. ^d^Based on 145 individuals

For Aim 3, we conducted hierarchical ordinal logistic regression to examine the association between ICBS, ICS, and stress scale and MOUD effectiveness, acceptability, and training. The ICBS, ICS, and stress scale did not predict any MOUD effectiveness or acceptability. ICBS predicted each MOUD outcome when examining training. For example, for each unit increase in ICBS, the cumulative logit of being in the lowest response category (i.e., no training) for methadone training decreased by 0.96 (95% CI -1.87, -0.05). Stated differently, with greater endorsement on the ICBS scale, the likelihood of endorsing less methadone training decreased. (Table 4).

**Table 4:** Results from Cross-Sectional Hierarchical Ordinal Logistic Regression using Multiply Imputed Dataset

	Parameter estimate (95% CI)				
	Effectiveness				
**Predictor**	Methadone	Bup (oral)	Bup (inject)	Nal (oral)	Nal (inject)
Implementation Citizenship Behavior Scale	-0.17 (-0.91, 0.57)	-0.15 (-0.97, 0.67)	-0.49 (-0.37, 0.38)	-0.09 (-1.00, 0.82)	-0.18 (-1.33, 0.98)
Implementation Climate Scale	-0.79 (-1.82, 0.25)	-0.54 (-1.41, 0.32)	-0.94 (-2.17, 0.29)	-0.24 (-1.42, 0.94)	-0.64 (-2.25, 0.96)
Stress Scale	0.02 (-0.07, 0.12)	-0.01 (-0.08, 0.07)	0 (-0.16, 0.16)	0 (-0.08, 0.08)	0.01 (-0.22, 0.23)
	**Acceptability**				
Implementation Citizenship Behavior Scale	-0.23 (-1.01, 0.54)	-0.19 (-0.91, 0.54)	-0.03 (-0.63, 0.86)	-0.04 (-0.92, 0.84)	-0.03 (-1.02, 0.96)
Implementation Climate Scale	-0.57 (-1.68, 0.54)	-0.38 (-1.40, 0.64)	-0.51 (-1.79, 0.78)	-0.49 (-1.84, 0.87)	-0.77 (-2.29, 0.76)
Stress Scale	0.01 (-0.08, 0.11)	0.02 (-0.06, 0.09)	0.06 (-0.02, 0.13)	0 (-0.06, 0.07)	0.03 (-0.03, 0.10)
	**Training**				
Implementation Citizenship Behavior Scale	-0.96 (-1.87, -0.05)* ^a^	-1.03 (-1.80, -0.26)**	-1.03 (-1.99, -0.06)*	-1.02 (-1.88, -0.15)*	-0.97 (-1.81, -0.13)*
Implementation Climate Scale	-0.41 (-1.44, 0.61)^a^	-0.46 (-1.48, 0.56)	-0.17 (-1.37, 1.04)	-0.33 (-1.51, 0.83)	-0.46 (-1.75, 0.83)
Stress Scale	0.04 (-0.03, 0.10)^a^	-0.01 (-0.08, 0.07)	-0.01 (-0.09, 0.08)	-0.01 (-0.07, 0.06)	0 (-0.07, 0.08)

*Note* Probabilities of lowered ordered categories (i.e., lower endorsed effectiveness, acceptability, training) were modeled. * *p* < 0.05. ** *p* < 0.01 (significant bolded). Based on 1050 observations (to 105 individuals). Includes covariate adjustment of system (YLS or CMHC), age, gender, race, ethnicity, education level, time at position, job satisfaction, and rurality. ^a^Between-imputation variance was zero for the predictors; parameter estimates were equivalent across imputations; results from 10th imputation presented

## Discussion

The current paper aimed to characterize attitudes toward and training in MOUDs among those who interact professionally with youth. Specifically, we aimed to determine whether MOUDs differ from one another, whether MOUD ratings differed by demographic characteristics, and the association between workplace-related variables and MOUDs.

### Aim 1: Differences between MOUD type

First, numerous important findings emerged when comparing attitudes about and training in different types of MOUD. Results indicated that 22 out of the 30 analyses (10 comparisons each for effectiveness, acceptability, and training) suggested statistically significant differences. Acceptability and effectiveness demonstrated similar results, such that there were no statistically significant differences demonstrated for type of buprenorphine (oral versus injection). Therefore, attitudes toward buprenorphine may be similar regardless of routes of administration. However, buprenorphine training differed by route of administration; individuals reported receiving more oral buprenorphine training compared to every other MOUD.

Interestingly, methadone was viewed as the least effective or acceptable MOUD, which is consistent with prior research among counselors at substance use treatment centers [31]. However, respondents had more training in methadone compared to all MOUDs except oral buprenorphine. This study cannot establish directionality in the relationship between attitudes and training and, therefore, cannot illuminate this paradoxical finding, as we would expect training facilitates the increase in positive attitudes towards interventions. This finding may also reflect a cohort effect, as methadone has been regulated for OUD since the 1970s and approximately 60% of the sample is over age 36 and 33% over age 46. Respondents may be more familiar with methadone and may continue to hold more negative attitudes about methadone compared to other MOUDs. Despite daily administration with behavioral supports, research continues to support that the general public [52], OUD patients [53], and providers [42] hold more negative views toward methadone compared to buprenorphine and naltrexone.

While methadone demonstrated reduced acceptability and perceived effectiveness compared to buprenorphine and naltrexone, attitudes toward buprenorphine and naltrexone appeared to differ based on route of administration, where injection was viewed as more effective and acceptable than oral. While prior research has demonstrated the limited effectiveness of oral naltrexone with regard to opioid abstinence and treatment engagement in applied settings [54], we are not aware of prior research that has investigated perceived acceptability and effectiveness of MOUD by route of administration among CMHC and YLS respondents; future research is needed to replicate this finding.

### Aim 2: Difference between MOUD and demographic variables

For the second aim, group differences were explored by MOUD. System, rurality, age, gender, and education level explained group differences in effectiveness, and age and education level explained differences in training. Notably, there were system-level differences in methadone and buprenorphine; CMHC employees reported greater perceived effectiveness of methadone and buprenorphine (both oral and injectable) than YLS employees. This is consistent with prior research demonstrating particularly negative MOUD attitudes among legal system employees [55]. These differences in attitudes may be due to numerous possible explanations, such as the nature of interactions with populations presenting with substance use concerns, stigmatizing beliefs of substance use disorders, and minimal resources to obtain ongoing professional training. Respondents employed in more rural counties rated oral buprenorphine as more effective than those in less rural counties. Prior research presents a conflicting picture about MOUD attitudes and rurality. Prior research that has examined rural-specific MOUD barriers identified both availability and acceptability barriers; there are few MOUD providers and providers have negative attitudes about substance use intervention [46]. However, other research has found that substance use treatment clinic providers in rural clinics endorsed more positive perceptions of naltrexone than less rural providers [30], and non-prescribing providers in rural communities endorsed more favorability of MOUD-only treatment compared to combined medication and psychosocial interventions [35]. The role of rurality on MOUD attitudes may be dependent on numerous factors (e.g., access/availability) not examined in the current study.

While gender is crudely estimated in the current study and likely interacts with a host of other factors (e.g., employment in CMHC vs. YLS, education level, stigmatizing beliefs), our results found a significant difference by gender when examining perceived effectiveness of injectable naltrexone. Post hoc pairwise comparisons did not reveal significant differences, potentially explained by low power. Prior research has demonstrated that women endorsed more favorable views toward EBPs than men [49]. Ongoing research is needed to understand the interaction between various identities to understand why such group identities may facilitate or hinder MOUD beliefs and training.

Finally, age and education level differentiated perceived effectiveness and training in various MOUDs. No clear pattern emerged by MOUD type, and results are best summarized as early and middle career professionals (aged 36–45 years) perceived certain MOUDs as more effective and had more training in certain MOUDs than younger and older age groups. We did not have measures of the amount or type of MOUD training; however, we suspect that this 36- to 45-year-old age group is correlated with professional experience and continuing education related to substance use disorder interventions (including medications). Prior research has demonstrated associations among age, education, and attitudes toward MOUDs. For example, providers working in substance use treatment facilities who had more advanced degrees endorsed greater perceived effectiveness of methadone, buprenorphine, and naltrexone. Of note, apart from gender, attitudes toward naltrexone did not differ by demographic variables. Our observed null results for naltrexone may indicate insufficient power and/or be reflective of minimal knowledge or training about this medication among a primarily youth-serving workforce.

### Aim 3: MOUD prediction from workplace variables

When examining associations between workplace variables and MOUDs, the Implementation Citizenship Behavior Scale (ICBS) predicted training in each MOUD, such that greater ICBS endorsement was associated with more endorsed MOUD training. This finding attests to the importance of individual characteristics and attitudes (to pursue EBPs) in actual practice. However, there may exist a sequence between organizational climate promoting individual initiatives, which may in turn be related to EBPs training. Or, perhaps, organization climate may moderate the relationship between personal initiatives to implement EBPs and EBP training. Such analyses were not possible given the cross-sectional data. Future studies that explore the relationship among organizational and individuals support of EBPs would be beneficial to strengthen understanding of multi-level interventions to promote MOUD uptake.

Additionally, it is worth noting that no workplace variables were significantly associated with perceptions of the effectiveness or acceptability of MOUDs. It is uncertain whether the sample is underpowered to detect such effects, or these workplace variables are unrelated to MOUD attitudes. Most research investigating MOUD attitudes calls for an increase in MOUD training, or mental health training more broadly [45]. However, much research is needed to determine what training and for whom is most potent in changing MOUD attitudes, and relatedly, how MOUD attitudes impact messages delivered to legal-involved youth.

### Strengths and limitations

The current study was strengthened by examining MOUDs across domains (i.e., attitudes toward effectiveness and acceptability, and training) and their relationship to numerous demographic and workplace characteristics. Additionally, past research on MOUDs is focused on health care professionals’ attitudes, with minimal research on employees within the YLS. The combination of both YLS and CMHC employees also allowed for comparing by system.

Despite these strengths, the study was limited by a few key factors. First, we lacked specificity in the questionnaire about attitudes toward and training in MOUDs *for youth*. Prior research has demonstrated that attitudes toward MOUDs differ based on whether questions pertained to youth or adults [14]. As examples, oral naltrexone prescribing patterns may differ between adults and youth, as the adult addiction workforce is comparatively naïve to oral naltrexone, while prescribers to youth may be more comfortable with daily observed oral dosing in youth who have caregiver supervision. Methadone is also rarely prescribed for adolescents and, therefore, may not be viewed as acceptable for youth. It is important to investigate attitudes towards and training in MOUDs when referencing treatment for youth versus adults.

Second, the sample of respondents was predominantly a White, Non-Hispanic population. Beliefs about MOUDs and messages conveyed to youth may differ based on respondent race (and its interaction with youth race). Given the homogenous sample in terms of race and ethnicity, we grouped individuals identifying with the non-majority group into one group, which limits our understanding within and across minority groups.

Third, we did not collect information from survey respondents about their lived experience with opioid use. Attitudes toward MOUDs may be dependent on personal experience with opioid use [44] and would be important to consider in future studies. Relatedly, while data for the current study were derived from a larger mixed-methods study, we did not conduct qualitative interviews to understand respondents’ views toward MOUDs, which may have offered clarification regarding attitudes toward specific MOUDs and prior MOUD training. Combining quantitative analysis with follow-up qualitative interviews will advance the field in elucidating potential mechanisms to change MOUD attitudes, or attitudes toward substance use disorders, more broadly.

Finally, we did not investigate how attitudes and training are related to messaging provided to youth and treatment referrals. Prior research has suggested that both theories of reasoned action and planned behavior predicted whether substance use treatment providers recommended medication for substance use disorders [56]; such theories may aid interventions with key stakeholders.

## Conclusions

The current study investigated attitudes toward and training in MOUDs among employees in the YLS and CMHCs who interface with youth professionally. Using cross-sectional survey data from 181 respondents, we found that MOUDs largely differed from one another; system (YLS vs. CMHC), rurality age, gender, and education level explained differences in perceived effectiveness and training in certain MOUDs; and personal initiatives to support and implement EBPs in one’s workplace were associated with MOUD training. The results establish descriptive analyses to help direct future intervention studies to facilitate positive attitudes toward and uptake of MOUDs. We recommend future studies consider examining MOUD by route of administration to examine the replicability of our results, as well as examine the predictors or and mediators of MOUD attitudes in longitudinal studies. Notably, the role of organizational factors, lived experience with opioid use, and MOUD training are important factors to examine in relation to MOUD attitudinal change.

### Electronic supplementary material

Below is the link to the electronic supplementary material.

Supplementary Material 1

## Data Availability

No datasets were generated or analysed during the current study.

## Abbreviations

MOUD: Medication for opioid use disorder
OUD: Opioid use disorder
EBP: Evidence-based practice
CMHC: Community mental health centers
YLS: Youth legal system
ADAPT: Alliances to Disseminate Addiction Prevention and Treatment
ICBS: Implementation Citizenship Behavior Scale
ICS: Implementation Climate Scale
ANOVA: Analysis of variance
CI: Confidence interval
