# Supplementary material for: Attitudes toward and training in medications for opioid use disorders: a descriptive analysis among employees in the youth legal system and community mental health centers
Source: Subst Abuse Treat Prev Policy. 2024 Jun 21;19:32. doi: 10.1186/s13011-024-00614-w (PMC11193280; doi:10.1186/s13011-024-00614-w)
Supplement: Supplementary file 1 — Supplementary Material 1 [file 13011_2024_614_MOESM1_ESM.docx]

Appendix.

Appendix 1. Sample Characteristics.

Appendix 2. Univariate statistics od MOUD attitudes and training.

Appendix 3. Results from Kruskal-Wallis Tests of Group Differences in Acceptability of MOUDs by Demographic Variables.

| Appendix 1. Sample characteristics. | | |
| --- | --- | --- |
|  | N (%)^a^ | |
|  | Full sample (n=181) | Multiple imputed sample (n=105) |
| System |  |  |
| CMHC | 119 (65.8) | 70 (66.7) |
| YLS | 62 (34.3) | 35 (33.3) |
| Rurality |  |  |
| More rural | 44 (24.7) | 27 (25.7) |
| Less rural | 134 (75.3) | 78 (74.3) |
| Age |  |  |
| 18-25 | 16 (8.8) | 4 (3.8) |
| 26-35 | 53 (29.3) | 29 (27.6) |
| 36-45 | 51 (28.2) | 37 (35.2) |
| 46-55 | 36 (19.9) | 23 (21.9) |
| 56-65 | 20 (11.1) | 9 (8.6) |
| >65 | 4 (2.2) | 3 (2.9) |
| Unknown | 1 (0.6) | - |
| Gender |  |  |
| Male | 36 (19.9) | 21 (20.0) |
| Female | 141 (77.9) | 81 (77.1) |
| Other | 4 (2.2) | 3 (2.9) |
| Race |  |  |
| Asian | 4 (2.2) | 2 (1.9) |
| Black/African American | 3 (1.7) | 2 (1.9) |
| Multiracial | 2 (1.1) | 2 (1.9) |
| White | 167 (92.3) | 98 (93.3) |
| Unknown | 5 (2.8) | 1 (0.95) |
| Ethnicity |  |  |
| Hispanic | 9 (5.0) | 5 (4.8) |
| Non-Hispanic | 167 (92.3) | 100 (95.2) |
| Unknown | 5 (2.8) | - |
| Time at Position |  |  |
| <1 year | 27 (14.9) | 12 (11.4) |
| 1-4 years | 94 (51.9) | 57 (54.3) |
| 5-9 years | 26 (14.4) | 17 (16.2) |
| 10-14 years | 11 (6.1) | 8 (7.6) |
| 15-19 years | 12 (6.6) | 5 (4.8) |
| >19 years | 10 (5.5) | 6 (5.7) |
| Unknown | 1 (0.6) | - |
| Job Satisfaction |  |  |
| Very dissatisfied | 2 (1.1) | 0 |
| Dissatisfied | 6 (3.3) | 4 (3.8) |
| Not satisfied/dissatisfied | 20 (11.1) | 10 (9.5) |
| Satisfied | 99 (54.7) | 58 (55.2) |
| Very satisfied | 53 (29.3) | 33 (31.4) |
| Unknown | 1 (0.6) | - |
| Education Level |  |  |
| Some college | 6 (3.3) | 3 (2.9) |
| Bachelor’s | 89 (49.2) | 46 (43.8) |
| Associate’s | 3 (1.7) | 2 (1.9) |
| Master’s | 71 (39.2) | 45 (42.9) |
| Doctorate | 11 (6.1) | 9 (8.6) |
| Unknown | 1 (0.6) | - |
| ^a^Percentages rounded to the nearest tenth. | | |

| Appendix 2. Univariate statistics of MOUD attitudes and training. | | | | | | | |
| --- | --- | --- | --- | --- | --- | --- | --- |
|  | Effectiveness | | | | | | |
|  | N | Mean (SD) | Median | Mode | Variance | Skewness | Kurtosis |
| Methadone | 122 | 4.18 (1.83) | 4 | 5 | 3.34 | -0.21 | -0.94 |
| Buprenorphine (oral) | 120 | 4.77 (1.67) | 5 | 5 | 2.80 | -0.48 | -0.50 |
| Buprenorphine (inject) | 98 | 4.78 (0.77) | 5 | 5 | 3.14 | -0.58 | -0.39 |
| Naltrexone (oral) | 99 | 4.48 (1.70) | 5 | 5 | 2.88 | -0.41 | -0.48 |
| Naltrexone (inject) | 103 | 5 (1.73) | 5 | 5 | 2.98 | -0.82 | 0.05 |
|  |  |  |  |  |  |  |  |
|  | Acceptability | | | | | | |
| Methadone | 126 | 4.42 (1.83) | 5 | 5 | 3.35 | -0.24 | -0.89 |
| Buprenorphine (oral) | 124 | 4.90 (1.81) | 5 | 7 | 3.27 | -0.62 | -0.53 |
| Buprenorphine (inject) | 113 | 4.98 (1.84) | 5 | 7 | 3.39 | -0.65 | -0.51 |
| Naltrexone (oral) | 111 | 4.95 (1.81) | 5 | 7 | 3.28 | -0.71 | -0.30 |
| Naltrexone (inject) | 117 | 5.27 (1.76) | 6 | 7 | 3.11 | -0.96 | 0.20 |
|  |  |  |  |  |  |  |  |
|  | Training | | | | | | |
| Methadone | 146 | 2.88 (1.93) | 2 | 1 | 3.72 | 0.60 | -0.90 |
| Buprenorphine (oral) | 144 | 2.97 (2.03) | 2 | 1 | 4.13 | 0.64 | -0.91 |
| Buprenorphine (inject) | 144 | 2.47 (1.88) | 2 | 1 | 3.52 | 1.02 | 0.01 |
| Naltrexone (oral) | 144 | 1.97 (0.93) | 2 | 1 | 3.89 | 0.93 | -0.45 |
| Naltrexone (inject) | 143 | 2.69 (2.00) | 2 | 1 | 4.01 | 0.89 | -0.54 |

| Appendix 3. Results from Kruskal-Wallis Tests of Group Differences in Acceptability of MOUDs by Demographic Variables. | | | | | | | | | | |
| --- | --- | --- | --- | --- | --- | --- | --- | --- | --- | --- |
|  | Methadone^a^ | | Bup (oral)^b^ | | Bup (inject)^c^ | | Nal (oral)^d^ | | Nal (inject)^e^ | |
| **Variable** | Mean of Ranks Sums (N) | (χ^2^, df,  p-value) | Mean of Ranks Sums (N) | (χ^2^, df,  p-value) | Mean of Ranks Sums (N) | (χ^2^, df,  p-value) | Mean of Ranks Sums (N) | (χ^2^, df, p-value) | Mean of Ranks Sums (N) | (χ^2^, df,  p-value) |
| System |  |  |  |  |  |  |  |  |  |  |
| YLS | 62.9 (41) | 0.02 (1), p=0.895 | 59.7 (39) | 0.36 (1), p=0.550 | 51.9 (36) | 1.32 (1), p=0.251 | 51.5 (35) | 1.05 (1), p=0.305 | 56.3 (37) | 0.36 (1), p=0.546 |
| CMHC | 63.8 (85) |  | 63.8 (85) |  | 59.4 (77) |  | 58.1 (76) |  | 60.3 (80) |  |
| Rurality |  |  |  |  |  |  |  |  |  |  |
| Less rural | 60.7 (30) | 0.10 (1), p=0.749 | 59.4 (30) | 0.150 (1), p=0.699 | 54.4 (28) | 0.09 (1), p=0.761 | 59.7 (27) | 0.84 (1), p=0.360 | 57.4 (30) | 0.01 (1), p=0.909 |
| More rural | 63.1 (94) |  | 92.2 (92) |  | 56.5 (83) |  | 53.4 (82) |  | 58.2 (85) |  |
| Age |  |  |  |  |  |  |  |  |  |  |
| 18-25 | 45.9 (5) | 5.84 (5), p=0.322 | 45.6 (6) | 5.86 (5), p=0.320 | 27.3 (3) | 9.81 (5), p=0.081 | 38.4 (4) | 4.27 (5), p=0.512 | 34.3 (4) | 4.39 (5), p=0.495 |
| 26-35 | 70.8 (38) |  | 68.2 (36) |  | 59.5 (33) |  | 54.4 (31) |  | 57.0 (33) |  |
| 36-45 | 67.3 (41) |  | 67.2 (41) |  | 63.4 (40) |  | 62.4 (37) |  | 65.8 (38) |  |
| 46-55 | 56.2 (28) |  | 58.4 (27) |  | 52.0 (25) |  | 56.3 (26) |  | 55.7 (28) |  |
| 56-65 | 49.7 (10) |  | 45.2 (10) |  | 34.1 (8) |  | 44.1 (10) |  | 57.2 (10) |  |
| 66+ | 63.4 (4) |  | 59.3 (4) |  | 71.1 (4) |  | 55.3 (3) |  | 63.6 (4) |  |
| Gender |  |  |  |  |  |  |  |  |  |  |
| Male | 61.5 (97) | 3.06 (2), p=0.217 | 60.1 (96) | 3.05 (2), p=0.217 | 57.2 (87) | 2.20 (2), p=0.333 | 54.3 (88) | 3.84 (2), p=0.147 | 58.5 (92) | 2.79 (2), p=0.248 |
| Female | 67.3 (26) |  | 68.6 (26) |  | 53.0 (23) |  | 59.2 (21) |  | 57.8 (23) |  |
| Other | 96.0 (3) |  | 96.5 (2) |  | 82.2 (3) |  | 97.0 (2) |  | 97.5 (2) |  |
| Race |  |  |  |  |  |  |  |  |  |  |
| White | 63.9 (116) | 0.15 (1), p=0.697 | 62.1 (114) | 0.15 (1), p=0.702 | 57.2 (103) | 0.03 (1), p=0.865 | 56.3 (101) | 0.14 (1), p=0.713 | 60.0 (107) | 1.21 (1), p=0.271 |
| Racial minority | 59.3 (10) |  | 66.6 (10) |  | 55.4 (10) |  | 52.5 (10) |  | 48.1 (10) |  |
| Ethnicity |  |  |  |  |  |  |  |  |  |  |
| Non-Hispanic | 62.5 (119) | 0.65 (1), p=0.422 | 61.4 (115) | 1.95 (1), p=0.162 | 56.3 (105) | 1.60 (1), p=0.206 | 56.7 (105) | 3.30 (1), p=0.069 | 58.7 (110) | 1.18 (1), p=0.277 |
| Hispanic | 48.1 (4) |  | 39.6 (5) |  | 38.2 (5) |  | 30.7 (5) |  | 42.6 (5) |  |
| Time at Position |  |  |  |  |  |  |  |  |  |  |
| <1 year | 74.3 (16) | 3.71 (3), p=0.295 | 68.9 (17) | 1.73 (3), p=0.631 | 67.8 (12) | 1.55 (3), p=0.671 | 60.7 (11) | 0.817 (3), p=0.846 | 64.2 (13) | 0.72 (3), p=0.869 |
| 1-4 years | 63.4 (67) |  | 60.5 (66) |  | 55.9 (63) |  | 54.4 (61) |  | 56.9 (63) |  |
| 5-9 years | 51.8 (20) |  | 57.4 (19) |  | 54.4 (19) |  | 54.1 (19) |  | 59.5 (19) |  |
| 10+ years | 66.6 (23) |  | 68.1 (22) |  | 56.4 (19) |  | 60.2 (20) |  | 61.5 (22) |  |
| Education |  |  |  |  |  |  |  |  |  |  |
| Some college | 41.6 (4) | 2.05 (4), p=0.726 | 71.9 (4) | 1.73 (4), p=0.785 | 52.3 (2) | 0.75 (4), p=0.945 | 59.7 (3) | 1.55 (4), p=0.818 | 63.5 (3) | 1.11 (4), p=0.892 |
| Bachelor’s | 62.2 (55) |  | 58.6 (52) |  | 54.4 (47) |  | 52.6 (48) |  | 55.7 (50) |  |
| Associate’s | 60.5 (2) |  | 57.0 (2) |  | 50.8 (2) |  | 51.0 (2) |  | 51.0 (2) |  |
| Master’s | 65.3 (55) |  | 64.1 (56) |  | 59.5 (53) |  | 57.6 (49) |  | 61.3 (52) |  |
| Doctorate | 70.2 (10) |  | 71.4 (10) |  | 58.2 (9) |  | 65.2 (9) |  | 63.5 (10) |  |
| Job Satisfaction |  |  |  |  |  |  |  |  |  |  |
| Dissatisfied | 80.6 (4) | 1.03 (2), p=0.598 | 69.3 (4) | 0.39 (2), p=0.822 | 66.3 (2) | 0.70 (2),  p=0.705 | 55.6 (4) | 0.07 (2), p=0.964 | 62.4 94) | 0.98 (2), p=0.613 |
| Neutral | 60.3 (15) |  | 66.8 (13) |  | 51.7 (13) |  | 53.7 (12) |  | 50.6 (13) |  |
| Satisfied | 63.3 (107) |  | 61.7 (107) |  | 57.3 (96) |  | 56.3 (95) |  | 60.0 (100) |  |
| Note: p<0.05 is bolded. Mean scores are derived from sum of rank sums/N. ^a^Based on 126 individuals. ^b^Based on 124 individuals. ^c^Based on 113 individuals. ^d^Based on 111 individuals. ^e^Based on 117 individuals. | | | | | | | | | | |
